# Effects of Acupuncture on Chronic Stress-Induced Depression-Like Behavior and Its Central Neural Mechanism

**DOI:** 10.3389/fpsyg.2019.01353

**Published:** 2019-07-05

**Authors:** Min-Ju Lee, Jae-Sang Ryu, Seul-Ki Won, Uk Namgung, Jeeyoun Jung, So-Min Lee, Ji-Yeun Park

**Affiliations:** ^1^ Department of Korean Medicine, College of Korean Medicine, Daejeon University, Daejeon, South Korea; ^2^ Clinical Medicine Division, Korea Institute of Oriental Medicine, Daejeon, South Korea

**Keywords:** acupuncture, brain neural activity, chronic restraint stress, depressive-like behavior, serotonin receptor modulation

## Abstract

Depression is a serious psychiatric disorder with an enormous socioeconomic burden, and it is commonly comorbid with pain, chronic fatigue, or other inflammatory diseases. Recent studies have shown that acupuncture is an effective therapeutic method for reducing depressive symptoms; however, the underlying mechanism remains unknown. In this study, we investigated the effects of acupuncture on chronic stress-induced depression-like behavior and its central neural mechanisms in the brain. We induced chronic restraint stress (CRS) in male C57BL/6 mice for 14 or 28 consecutive days. Acupuncture treatment was performed at KI10·LR8·LU8·LR4 or control points for 7 or 14 days. Depression-like behavior was assessed with the open field test. Then, brain neural activity involving c-Fos and serotonin-related mechanisms *via* the 5-HT1A and 5-HT1B receptors were investigated. Acupuncture treatment at KI10·LR8·LU8·LR4 points rescued the depressive-like behavior, while control points (LU8·LR4·HT8·LR2) and non-acupoints on the hips did not. Brain neural activity was changed in the hippocampus, cingulate cortex, motor cortex, insular cortex, thalamus, and the hypothalamus after acupuncture treatment. Acupuncture treatment increased expression of 5-HT1A receptor in the cortex, hippocampus, thalamus, and the hypothalamus, and of 5-HT1B in the cortex and thalamus. In conclusion, acupuncture treatment at KI10·LR8·LU8·LR4 was effective in alleviating the depressive-like behavior in mice, and this therapeutic effect was produced through central brain neural activity and serotonin receptor modulation.

## Introduction

Depression is a common mood disorder, which has a high mortality and recurrence rate (Reddy, 2010). According to a previous study, about 10% of the world’s population has major depressive disorder (Riolo et al., 2005), and continued depression can lead to suicide, sleep disorders, anorexia, anxiety, and gangrene (Hammen, 2005). Depression is not only a serious disease *per se* but also has a major impact on the occurrence of other conditions, such as pain (Bair et al., 2003; Goldenberg, 2010), chronic fatigue syndrome (Bram et al., 2018), neurodegenerative disease (Modrego and Ferrández, 2004), and inflammatory diseases (Baerwald et al., 2019). The etiology of depression involves genetic, environmental, socioeconomic, and stress-related factors (Dunn et al., 2015). As stress is emerging as a major cause of depression (Yang et al., 2015), there is an interest in developing treatment methods for depression and elucidating the pathological and neurobiological mechanism of stress-induced depression. However, much of the fundamental mechanisms of stress-induced depression have yet to be elucidated.

Although the cause of depression remains unclear, the brain is known to play a fundamental role in the mechanism of depression (Leuchter et al., 1997). Changes in brain neural circuits or a chemical imbalance in the brain are involved in the onset of depression (Palazidou, 2012). Recent brain imaging studies have identified that several brain regions (Pandya et al., 2012), such as the hippocampus, amygdala, and anterior cingulate cortex are involved in depression (Drevets et al., 2008; Eisch and Petrik, 2012). Hippocampal neurons have bilateral connections to the amygdala, and relay the signals to several brain areas (Fried et al., 1997; McEwen et al., 2016). It also receives synaptic inputs through the cingulate cortex. Hippocampal activity is affected by a stressed state and is involved in stress-induced depression (Krugers et al., 2010; Kim et al., 2015). Since hippocampal neurons can be altered by serotonergic and adrenergic inputs, as well as by the corticotropin-releasing hormone, many antidepressants are aimed at blocking or modulating serotonin or serotonin receptors (Kohler et al., 2016).

The serotonergic system is one of the major neurotransmitter systems involved in depression. Monoaminergic neurotransmission imbalance is causally related to the clinical features of depression, and depletion of monoamines such as serotonin (5-hydroxytryptamine, 5-HT) in the brain is a widely accepted hypothesis in the field of depression (Rot et al., 2009). Conventional antidepressants that improve 5-HT transmission or inhibit 5-HT reuptake, such as selective serotonin-reuptake inhibitors (SSRIs), are the most commonly used pharmacotherapy (Gijsman et al., 2004; Holtzheimer and Nemeroff, 2006). However, the efficacy of these drug in some patients remains controversial (Torrens et al., 2005; Papakostas et al., 2008), and they also induce side effects, such as a lack of concentration, gastrointestinal disorder, recurrence of depression, and involve the inconvenience of long-term administration (Penn and Tracy, 2012). Therefore, various alternative therapies for depression are sought.

Acupuncture has been used to treat various disorders, including pain (Vickers et al., 2012; Park et al., 2014), neurodegenerative disease (Cho et al., 2012; Park et al., 2017a), and psychological disorders, such as depression and stress-induced symptoms (Mukaino et al., 2005; Leo and Ligot, 2007), with a very rare occurrence of adverse events. Previous studies have reported that acupuncture is an effective therapeutic approach for improving symptoms of depression (Pilkington, 2010; Zhang et al., 2010; Qu et al., 2013; Chan et al., 2015; Li et al., 2018) by regulating the mTOR signal (Oh et al., 2018), glial glutamate transporter (Luo et al., 2017), neuropeptide Y (Eshkevari et al., 2012), and ERK-CREB pathways in the brain (Lu et al., 2013), as well as the expression of hippocampal brain-derived neurotrophic factor (Yun et al., 2002). However, the biological basis of its efficacy remains largely unknown. Most *in vivo* acupuncture studies aimed at elucidating the effect and the fundamental mechanism underlying acupuncture effects have used a single acupoint for therapy. ST36 has been most commonly used, while some studies used PC6, GB20, and EX-HN3 (Yun et al., 2002; Eshkevari et al., 2012; Lu et al., 2013, 2016). In the clinic, however, practitioners generally use a combination of acupoints, selected on the basis of traditional Korean or Chinese medical theory. Therefore, it is necessary to select acupuncture combinations that are frequently used in clinics when conducting efficacy evaluation and mechanistic studies.

In this study, we investigated the effect of acupuncture treatment consisting of a combination of specific acupoints (KI10·LR8·LU8·LR4) in a mouse model of chronic stress-induced depressive-like behavior. We also analyzed neural activation and the changes in 5-HT receptor expression in various brain regions, to elucidate the central neural mechanism of this acupuncture treatment.

## Materials And Methods

### Animals

Male C57BL/6 mice (age: 6–7 weeks old; weight: 20–25 g) were used in this study (Daehan Biolink, Eum-seong, Korea). Mice were acclimated for at least 1 week before the experiments and maintained with a 12-h light/dark cycle with free access to water and food. The animals were randomly divided, and five mice were placed in each cage (room temperature 25°C and humidity of 55%) in a clean room. Their weight was monitored every 2 days (Supplementary Figure S1). This study was carried out in accordance with recommendations in the guidelines for the Care and Use of Laboratory Animals of the National Institutes of Health. All experimental protocols used in this study were approved by the Institutional Animal Care and Use Committee (IACUC) at Daejeon University (approval no. DJUARB2016-40).

### Chronic Restraint Stress

All mice were exposed to chronic restraint stress (CRS). CRS was induced by placing mice individually into a 50-ml Falcon tube and the tubes were slightly tilted for 6 h per day (10:00–16:00), for 14 or 28 consecutive days to induce depressive-like behavior. Mice subjected to CRS could not access water and food during the CRS but could freely access water and food at the end of it.

### Behavioral Test

**Figure 1:**
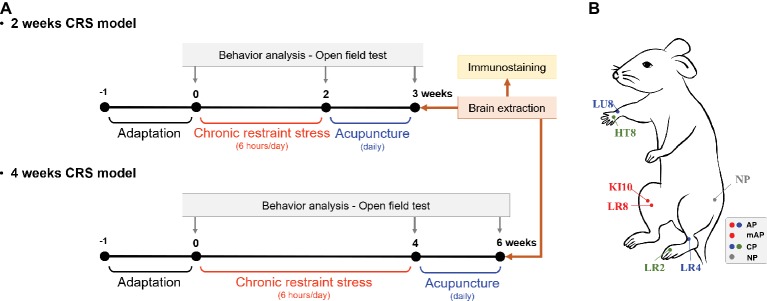
Experimental schedule and acupuncture treatment. **(A)** Two or 4 weeks of chronic restraint stress (CRS) was used to induce depressive-like behavior. Mice were treated with acupuncture for 7 or 14 consecutive days, respectively, and the open field test was performed to observe behavior patterns. **(B)** Location of the acupuncture points used in this study. AP, KI10·LR8·LU8·LR4; mAP, KI10·LR8; CP, LU8·LR4·HT8·LR2; NP, non-acupoints on the hips.

Mice were challenged in the open-field test (OFT) before and after the repeated restraint stress procedure to measure depressive-like behavior (Figure 1A). Mice were stabilized in the test room for more than 1 h prior to behavioral testing. Then, they were placed in a box (30 cm × 30 cm × 30 cm) which was made of white plastic and the total distance and number of zone transitions measured for a period of 10 min using a video camera system to track movement (SMART 3.0; Panlab S. L., Barcelona, Spain). The tracking images showed that the mice had locomotor activity and explorational ability. All the tests were performed between 10:00 am and 12:00 pm.

### Acupuncture Treatments

After CRS inducing, mice were randomly assigned to each group (*n* = 5–8 per group) according to the acupuncture treatment performed: CRS without acupuncture (CRS group), at the acupuncture points (KI10·LR8·LU8·LR4; AP group), at modified-acupuncture points (KI10·LR8; mAP group), at control points (LU8·LR4·HT8·LR2; CP group), or at non-acupoints (on the hips, located midway between the coccyx and hip joints; NP group) for 7 or 14 consecutive days. For acupuncture treatment, mice were slightly immobilized by fixing their neck. Then, acupuncture needles (0.18 mm in diameter and 8 mm in length; Dong-Bang Acupuncture Inc., Boryung, Korea) were inserted bilaterally at the appropriate positions according to group assignment. Needles were turned at a rate of two spins per second for 30 s, and then immediately removed (Yin et al., 2008). The CRS group was immobilized for the same amount of time to induce an equal amount of stress.

The location of each acupoint is as follows:

KI10 is located on the posteromedial aspect of the knee, just lateral to the semitendinosus tendon, in the popliteal crease. LR8 is located on the medial aspect of the knee, in the depression medial to the tendons of the semitendinosus and the semimembranosus muscles, at the medial end of the popliteal crease. LU8 is located on the anterolateral aspect of the forearm, between the radial styloid process and the radial artery, 1 cun superior to the palmar wrist crease. LR4 is located on the anteromedial aspect of the ankle, in the depression medial to the tibialis anterior tendon, anterior to the medial malleolus. HT8 is located on the palm of the hand, in the depression between the fourth and fifth metacarpal bones, proximal to the fifth metacarpophalangeal joint. LR2 is located on the dorsum of the foot, between the first and second toes, proximal to the web margin, at the border between the red and white flesh (Figure 1B; WHO, 2008; Yin et al., 2008).

### Brain Tissue Section Preparation

On the last day of the experiment, mice were anesthetized with an intraperitoneal injection of zoletil (30 mg/kg) and xylazine (10 mg/kg). Mice were transcardially perfused with 0.05 M phosphate-buffered saline (PBS) buffer followed by 4% paraformaldehyde (PFA). Their brains were removed, post-fixed overnight in cold 4% PFA, and then cryo-protected in a gradient of 10–30% sucrose solution until the brain sank. Brain tissues were subsequently cut into 40-μm-thick sections using a cryostat at −20°C (Leica Microsystems, Wetzlar, Germany), on the coronal plane, and sections were prepared in free-floating sections.

### Immunohistochemistry

Tissue sections were incubated in 1% H_2_O_2_ to reduce endogenous peroxidase activity, followed by incubation in 1% bovine serum albumin for 1 h at room temperature. Tissue sections were incubated with primary antibodies at 4°C overnight. The details of the primary antibodies were as follows: c-Fos monoclonal antibody (1:150, Santa Cruz, Dallas, TX, USA), 5-HT1A (1:100, Novus Biologicals, Centennial, CO, USA), and 5-HT1B (1:100, Abcam, Cambridge, UK). Thereafter, tissues were incubated with secondary antibodies at room temperature for 2 h; these included biotinylated goat anti-rabbit IgG (H + L) or anti-mouse IgG (H + L) (Vector Laboratories, Burlingame, CA, USA). After washing in PBS, tissue sections were incubated with ABC reagent (Vector Laboratories), with 0.02% diaminobenzidine and 0.003% hydrogen peroxide in 1 M Tris-buffered saline (pH 7.5). The tissues were then dehydrated by immersion in a gradient of 70–100% ethyl alcohol, followed by 100% xylene, before being mounted with Permount solution. Photographs of the stained brain sections were obtained under a microscope (Nikon, Minato, Japan).

The number of c-Fos positive cells on 30 brain regions of the cortex, cerebral nuclei, hippocampus, thalamus, hypothalamus, and midbrain, and the 5-HT1A- and 5-HT1B receptor-positive cells in 25 brain regions of the cortex, hippocampus, thalamus, and hypothalamus were manually counted, within a square of 32 μm × 32 μm. The mean values for the left and right regions were calculated and used for analysis. When the area was wider than the 32 μm × 32 μm square, three regions were randomly selected, and the average value was obtained. All measurements were randomly confirmed. All the brain regions assessed, and their abbreviations are shown in Table 1.

**Table 1:** Brain region and its abbreviation used in this study.

Region	Abbreviation
**Cortex**	
Cingulate cortex, area 1	CC-1
Cingulate cortex, area 2	CC-2
Primary motor cortex	MC-1
Secondary motor cortex	MC-2
Primary somatosensory cortex	SC-1
Secondary somatosensory cortex	SC-2
Agranular insular cortex, ventral part	IC-AIV
Insular cortex, dorsal part	IC-AID
Granular insular cortex	IC-Gi
Dysgranular insular cortex	IC-Di
Piriform cortex	Pir
**Cerebral nuclei**	
Caudate putamen (striatum) – dorsal medial	ST-DM
Caudate putamen (striatum) – dorsal lateral	ST-DL
Caudate putamen (striatum) – ventral medial	ST-VL
**Hippocampus**	
Field CA1 of hippocampus	HIP-CA1
Field CA2 of hippocampus	HIP-CA2
Field CA3 of hippocampus	HIP-CA3
Dentate gyrus	HIP-DG
**Thalamus**	
Paraventricular thalamic nucleus	TH-PV
Central medial thalamic nucleus	TH-CM
Mediodorsal thalamic nucleus	TH-MD
Ventral posterior thalamic nucleus	TH-VP
**Hypothalamus**	
Paraventricular hypothalamic nucleus	HyTH-PVN
Arcuate hypothalamic nucleus	HyTH-ARC
Ventromedial hypothalamic nucleus	HyTH-VM
Dorsomedial hypothalamic nucleus	HyTH-DM
Lateral hypothalamic area	HyTH-LH
Posterior hypothalamic area	HyTH-PH
**Midbrain**	
Dorsomedial periaqueductal gray	PAG-DM
Lateral periaqueductal gray	PAG-L

### Immunoblotting Assay

Proteins were extracted from the brain tissues using RIPA buffer containing a protease and phosphatase inhibitor. The protein amount was measured by a BCA protein assay kit (Thermo Fisher Scientific). Protein lysates (10 μg) were loaded into a 10% SDS-polyacrylamide gel electrophoresis (PAGE) and transferred on polyvinylidene difluoride (PVDF) membranes. Membranes were incubated overnight at 4°C with primary antibodies of c-Fos (1:1,000, Santa Cruz). The secondary antibodies of anti-mouse IgG HRP (1:5,000, Sigma-Aldrich) and anti-rabbit IgG HRP (1:5,000, Sigma-Aldrich) were used. The bands were detected with an ECL western blotting substrate kit (1:1 solution, GE Health Care). All the results were normalized to the respective GAPDH.

### Statistical Analyses

All data are expressed as mean ± standard error (SEM). The statistical data were analyzed by two-way ANOVA followed by Bonferroni post-tests or one-way ANOVA, followed by Newman-Keul’s post-tests using GraphPad Prism 5.0 (GraphPad Software Inc., CA, USA). The significance was set at *p* < 0.05 for all experiments.

## Results

### AP Acupuncture Treatment Improves CRS-Induced Depressive-Like Behavior

**Figure 2:**
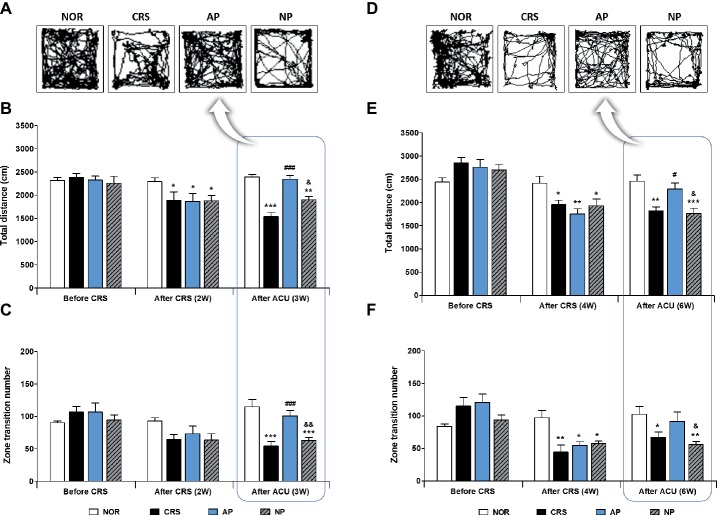
Acupuncture treatment at the AP (KI10·LR8·LU8·LR4) can reverse chronic restraint stress (CRS)-induced depressive-like behavior, as compared to non-acupoint treatment. Acupuncture treatment at KI10·LR8·LU8·LR4 significantly reversed the decrease in the total distance and the number of zone transitions caused by CRS (AP vs. CRS and non-acupoint treatment group) in both the 2-week **(A–C)** and 4-week CRS models **(D–F)**. NOR, normal; AP, CRS and acupuncture treatment at KI10·LR8·LU8·LR4; NP, CRS and acupuncture treatment at non-acupoints on the hips. *p < 0.05, **p < 0.01, ***p < 0.001 vs. NOR. ^#^*p* < 0.05, ^###^*p* < 0.001 vs. CRS. ^&^*p* < 0.05, ^&&^*p* < 0.01 vs. AP. Two-way ANOVA followed by the Bonferroni test. Error bars indicate SEM.

To investigate the anti-depressive effect of acupuncture treatment, we established a murine model of depressive-like behavior by 2 or 4 weeks of CRS. Then, acupuncture treatment, consisting of a specific combination of acupoints (KI10·LR8·LU8·LR4) was performed for 1 week (on the 2-week CRS model) or 2 weeks (on the 4-week CRS model). In the 2-week CRS model, CRS significantly decreased the total distance traveled and the zone transition number, as compared to the NOR mice (*p* < 0.001). Acupuncture treatment at the KI10·LR8·LU8·LR4 points significantly restored the total distance and the zone transition number as compared to the CRS group (*p* < 0.001). However, acupuncture treatment at the non-acupoints (NP group) did not have this rescue effect (Figures 2A–C). In the 4-week CRS model, acupuncture treatment at KI10·LR8·LU8·LR4 significantly rescued the decreased total distance as compared to the CRS and NP group (*p* < 0.05) (Figures 2D–F).

### Effects of Acupuncture Treatment at AP and mAP Points on 2 Weeks CRS-Induced Depressive-Like Behavior

**Figure 3:**
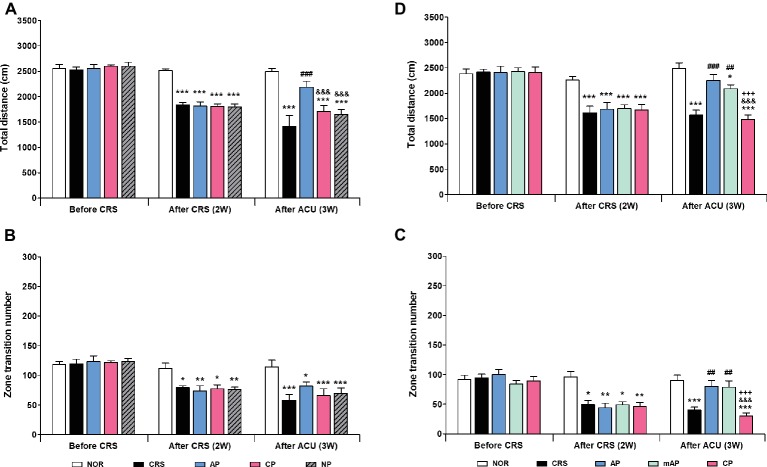
Acupuncture treatment at AP (KI10·LR8·LU8·LR4) and mAP (KI10·LR8) can reverse chronic restraint stress (CRS)-induced depressive-like behavior. **(A,B)** Acupuncture treatment at AP (KI10·LR8·LU8·LR4) significantly reversed the decrease in the total distance and the number of zone transitions caused by CRS (AP vs. CRS, CP, and NP treatment groups). Acupuncture treatment at AP and mAP significantly reversed the decrease in the total distance and the number of zone transitions compared to the CRS and CP treatment groups **(C,D)**. NOR, normal; AP, CRS and acupuncture treatment at KI10·LR8·LU8·LR4; mAP, CRS and acupuncture treatment at KI10·LR8; CP, CRS and acupuncture treatment at LU8·LR4·HT8·LR2; NP, CRS and acupuncture treatment at non-acupoints on the hips. *p < 0.05, **p < 0.01, ***p < 0.001 vs. NOR. ^##^*p* < 0.01, ^###^*p* < 0.001 vs. CRS. ^&&&^*p* < 0.001 vs. AP. ^+++^*p* < 0.001 vs. mAP. Two-way ANOVA followed by the Bonferroni test. Error bars indicate SEM.

Since AP treatment involved a combination of four acupoints and NP involved a single point, there was a possibility that the effect of AP treatment was more pronounced than NP treatment due to the intensity of the stimulation. Thus, we validated the therapeutic effect of AP in comparison to other combinations of four acupoints (CP; LU8·LR4·HT8·LR2). We found that AP treatment significantly rescued the decreased total distance (*p* < 0.001); the total distance traversed was significantly higher in the AP group than in the CP and NP groups (both *p* < 0.001). There was a similar trend in the zone transition number, but it did not reach statistical significance (Figures 3A,B).

Since acupuncture treatment at the AP (KI10·LR8·LU8·LR4) improved depression, while that at the CP (LU8·LR4·HT8·LR2) did not, we hypothesized that among the four acupoints in AP, KI10 and LR8 (mAP) were the major points involved in the therapeutic effect. As expected, the mAP group showed a significant improvement in depressive-like behavior (*p* < 0.01), similar to the AP group, and the AP while the mAP groups were significantly higher in both total travel distance and zone transition number than that of the CP group (both *p* < 0.001) (Figures 3C, D). This indicated that KI10·LR8·LU8·LR4, but particularly at KI10 and LR8, are the acupuncture points involved in the improvement of symptoms of depression.

### Acupuncture Treatment at AP Induces Brain Neural Activity in the CRS Model

To investigate the therapeutic mechanism of acupuncture, we analyzed how brain neural activity changed after acupuncture treatment. We used activation of the immediate early gene c-Fos as a marker of neuronal activity, according to previous studies (Chen et al., 2013; Wheeler et al., 2013). c-Fos activation was slightly lower in the hippocampus and hypothalamus of the 2-week CRS group, and AP increased the c-Fos activation in the hippocampus, thalamus, hypothalamus, and anterior cingulate cortex, as compared to the CRS group, but the difference was not statistically significant (Supplementary Figures S2A–D).

**Figure 4:**
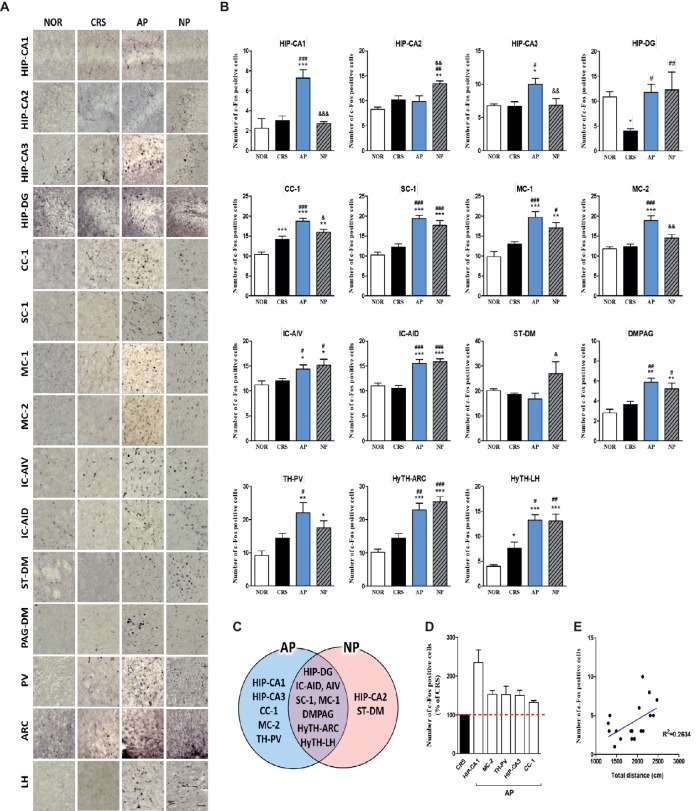
Acupuncture treatment at AP (KI10·LR8·LU8·LR4) causes changes in c-Fos expression in the brain in a 2-week CRS murine model. **(A,B)** Acupuncture treatment at AP (KI10·LR8·LU8·LR4) significantly increased c-Fos activation in specific regions of the cortex, cerebral nuclei, hippocampus, thalamus, hypothalamus, and midbrain. **(C)** Schematic Venn diagram representing the brain areas activated only by AP, only by NP, or by both stimuli. **(D)** Number of c-Fos-positive cells in brain regions changed only by acupuncture treatment (% of CRS group value). **(E)** Correlation between the c-Fos expression of HIP-CA1 and behavioral patterns in CRS, AP and NP group. NOR, normal; AP, CRS and acupuncture treatment at KI10·LR8·LU8·LR4; NP, CRS and acupuncture treatment at non-acupoints on the hips. *p < 0.05, **p < 0.01, ***p < 0.001 vs. NOR. ^#^*p* < 0.05, ^##^*p* < 0.01, ^###^*p* < 0.001 vs. CRS. ^&^*p* < 0.05, ^&&^*p* < 0.01, ^&&&^*p* < 0.001 vs. AP. One-way ANOVA followed by the Newman-Keuls test. Error bars indicate SEM.

For more detailed observations, we observed c-Fos activity by subdividing the cortex, cerebral nuclei, hippocampus, thalamus, hypothalamus, and the midbrain regions into a total of 30 regions. We observed significant changes in 15 of the 30 regions in the 2-week CRS model. c-Fos activation in the CA1 and CA3 of the hippocampus (HIP-CA1, HIP-CA3), cingulate cortex area 1 (CC-1), secondary motor cortex (MC-2), and paraventricular thalamic nucleus (TH-PV) was significantly increased after AP, but no other treatment. c-Fos activation in the dentate gyrus (HIP-DG), dorsal part of the insular cortex (IC-AID), ventral part of the insular cortex (IC-AIV), primary somatosensory cortex (SC-1), primary motor cortex (MC-1), arcuate nucleus of the hypothalamus (HyTH-ARC), lateral hypothalamic area (HyTH-LH), and dorsomedial periaqueductal gray (PAG-DM) was increased by both of AP and NP treatment. c-Fos activation in HIP-CA2 and the dorsomedial part of the striatum (ST-DM) was increased only after NP treatment (Figures 4A–C). Among the brain regions changed by AP, CA1 demonstrated the greatest change in comparison to the CRS group (Figure 4D). In the CRS, AP, and NP groups, c-Fos expression in CA1 showed a positive correlation with the results of the total distance travelled in the open field test (*p* < 0.05) (Figure 4E).

**Figure 5:**
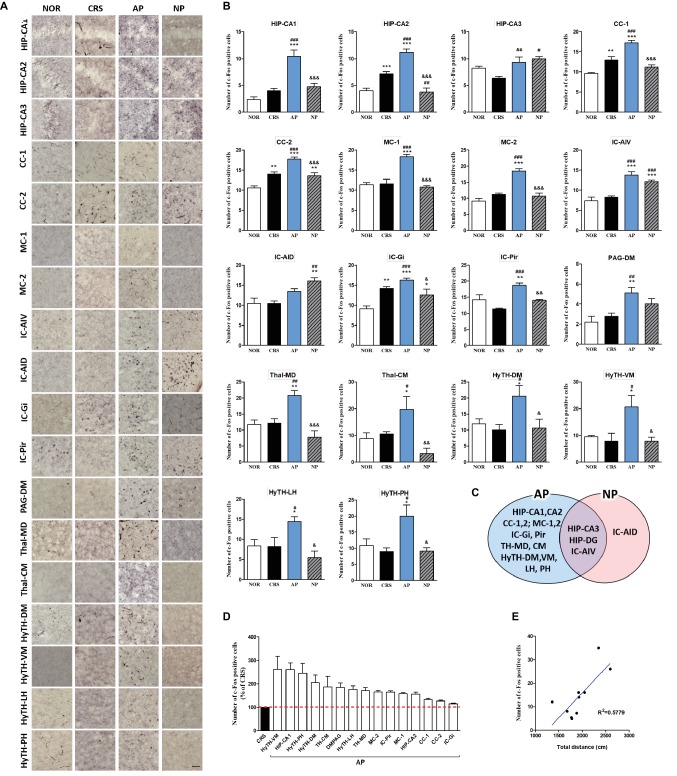
Acupuncture treatment at AP (KI10·LR8·LU8·LR4) causes changes in c-Fos expression in the brains of 4-week CRS model mice. **(A,B)** Acupuncture treatment at AP (KI10·LR8·LU8·LR4) significantly increased c-Fos activation in specific regions of the cortex, hippocampus, thalamus, and hypothalamus. **(C)** Schematic Venn diagram represents brain areas activated only by AP, only by NP, or by both stimuli. **(D)** Number of c-Fos-positive cells in brain regions changed only by acupuncture treatment (% of CRS group value). **(E)** Correlation between the c-Fos expression of HyTH-VM and behavioral patterns in CRS, AP, and NP groups. NOR, normal; AP, CRS and acupuncture treatment at KI10·LR8·LU8·LR4; NP, CRS and acupuncture treatment at non-acupoints on the hips. *p < 0.05, **p < 0.01, ***p < 0.001 vs. NOR. ^#^*p* < 0.05, ^##^*p* < 0.01, ^###^*p* < 0.001 vs. CRS. ^&^*p* < 0.05, ^&&^*p* < 0.01, ^&&&^*p* < 0.001 vs. AP. One-way ANOVA followed by the Newman-Keuls test. Error bars indicate SEM.

In the 4-week CRS model, c-Fos activation in the HIP-CA1, HIP-CA2, CC-1, cingulate cortex area 2 (CC-2), MC-1, MC-2, granular insular cortex (IC-Gi), piriform cortex (Pir), PAG-DM, mediodorsal thalamic nucleus (TH-MD), central medial thalamic nucleus (TH-CM), dorsomedial hypothalamic nucleus (HyTH-DM), ventromedial hypothalamic nucleus (HyTH-VM), HyTH-LH, and posterior hypothalamic area (HyTH-PH) was significantly increased only after AP treatment. c-Fos activation in the HIP-CA3 and IC-AIV was increased by both AP and NP treatment. c-Fos activation in the IC-AID was increased only after NP treatment (Figures 5A–C). Among the brain regions changed by AP treatment, HyTH-VM demonstrated the most change as compared to the CRS group (Figure 5D). In the CRS, AP, and NP groups, c-Fos expression in the HyTH-VM showed a positive correlation with the total distance travelled in the open field test (*p* < 0.01) (Figure 5E).

These results indicated that the cortex, hippocampus, thalamus, and the hypothalamus, which were the only regions changed by AP treatment, were the main regions mediating the therapeutic effect of acupuncture treatment.

### Regulation of 5-HT1A and 5-HT1B Receptors by AP Treatment

**Figure 6:**
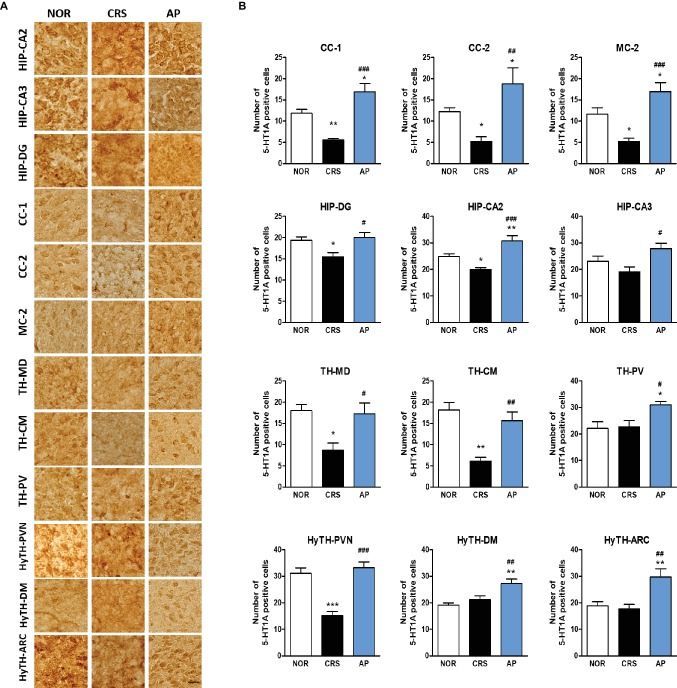
Acupuncture treatment at AP (KI10·LR8·LU8·LR4) changes 5-HT1A receptor expression in the cortex, hippocampus, thalamus, and hypothalamus. **(A,B)** Acupuncture treatment at AP (KI10·LR8·LU8·LR4) significantly increased 5-HT1A expression in the cortex, hippocampus, thalamus, and hypothalamus as compared to the CRS group. NOR, normal; AP, CRS and acupuncture treatment at KI10·LR8·LU8·LR4; NP, CRS and acupuncture treatment at non-acupoints on the hips. *p < 0.05, **p < 0.01, ***p < 0.001 vs. NOR. ^#^*p* < 0.05, ^##^*p* < 0.01, ^###^*p* < 0.001 vs. CRS. One-way ANOVA followed by the Newman-Keuls test. Error bars indicate SEM.

We further investigated whether acupuncture treatment at KI10·LR8·LU8·LR4 affected the expression of serotonin receptors. We analyzed the activation of 5-HT1A and 5-HT1B receptors in 25 brain regions of the cortex, hippocampus, thalamus, and the hypothalamus, which were identified as the main regions involved in mediating the effect of acupuncture on c-Fos expression. CRS significantly decreased 5-HT1A receptor activation in the CC-1, CC-2, MC-2, HIP-DG, HIP-CA2, TH-MD, TH-CM, and paraventricular hypothalamic nucleus (HyTH-PVN) as compared to the NOR group. Acupuncture treatment at KI10·LR8·LU8·LR4 significantly increased 5-HT1A receptor activation in the CC-1, CC-2, MC-2, HIP-DG, HIP-CA2, HIP-CA3, TH-MD, TH-CM, TH-PV, HyTH-PVN, HyTH-DM, and HyTH-ARC as compared to the CRS group (Figures 6A,B).

**Figure 7:**
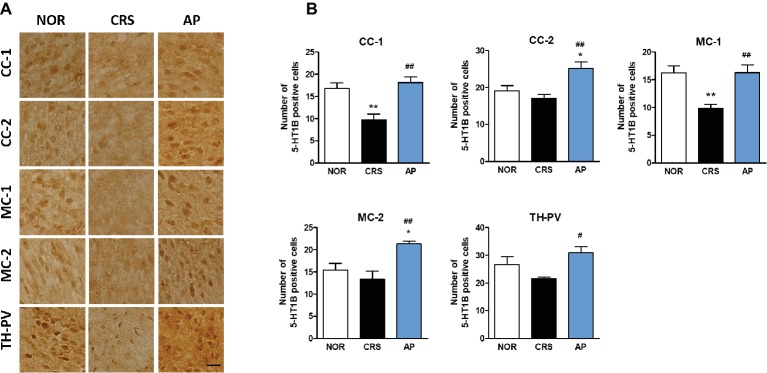
Acupuncture treatment at AP (KI10·LR8·LU8·LR4) changes 5-HT1B receptor expression in the cortex, hippocampus, thalamus, and hypothalamus. **(A,B)** Acupuncture treatment at AP (KI10·LR8·LU8·LR4) significantly increased 5-HT1B receptor expression in the cortex and thalamus as compared to the CRS group. NOR, normal; AP, CRS and acupuncture treatment at KI10·LR8·LU8·LR4; NP, CRS and acupuncture treatment at non-acupoints on the hips. *p < 0.05, **p < 0.01 vs. NOR. ^#^*p* < 0.05, ^##^*p* < 0.01 vs. CRS. One-way ANOVA followed by the Newman-Keuls test. Error bars indicate SEM.

CRS significantly decreased 5-HT1B receptor activation in the CC-1 and MC-1 compared to the NOR group. AP treatment significantly increased 5-HT1B receptor activation in the CC-1, CC-2, MC-1, MC-2, and TH-PV as compared to the CRS group (Figures 7A,B).

These results indicate that the effect of acupuncture treatment was mediated *via* central brain neural activity and modulation of serotonin receptors.

## Discussion

To the best of our knowledge, this study is the first to identify the effect of acupoint combination of KI10·LR8·LU8·LR4 in chronic stress-induced depressive-like behavior and its neural mechanism. In this study, we found that acupuncture treatment at KI10·LR8·LU8·LR4 was effective in improving CRS-induced depressive-like behavior, and that among these four acupoints, KI10 and LR8 were the major acupoints effective in the treatment of depressive-like symptoms. Moreover, we also found that this behavioral improvement was mediated by brain neuronal activity involving the modulation of serotonin receptor expression in specific brain regions.

Sa-am acupuncture is a unique Korean traditional acupuncture method developed by Sa-am in the seventeenth century A.D. and most widely adopted by Korean medical doctors in the clinic (Han et al., 2005). Sa-am acupuncture uses *five-transporting points* according to the principle of tonification and sedation. The combination of KI10·LR8·LU8·LR4, called Liver-tonification, has been reported as the most commonly used acupoint combination in Sa-am acupuncture (Yoon et al., 2018), and is used to treat a variety of pain and psychological conditions, such as depression (Kim, 2006; Yoon et al., 2018). However, despite being widely used clinically, few studies have reported the effect of the KI10·LR8·LU8·LR4 acupoint combination, and no animal studies to date have identified its therapeutic effects and underlying biological mechanisms.

In our study, we found that the acupuncture treatment at KI10·LR8·LU8·LR4 significantly improved depressive-like behavior in mice, as compared to non-acupoint treatment on the hips and at control points (LU8·LR4·HT8·LR2). The LU8·LR4·HT8·LR2 combination has been reported to be effective in reducing anxiety (Kim, 1998, 2006; Yoon et al., 2018), which shows a different pathological phenotype to that of depression. This combination also involves four acupoints and can thus exert the same amount of stimulation as the AP combination. Therefore, the difference in effectiveness between the two groups is not due to the amount of stimulation, but due to specific acupoints. We demonstrated that KI10·LR8·LU8·LR4, and particularly KI10 and LR8, play a pivotal role in improving depressive-like behavior. In order to establish a more precise basis in future, it will be necessary to study the therapeutic efficacy of each acupuncture point specifically and define the related mechanism.

To elucidate the therapeutic mechanisms of depression, researchers have focused on the brain, which controls all sensory and behavioral modulations (MacPherson et al., 2016). Since previous studies have reported that hippocampal atrophy occurs with depression (Bremner et al., 2000), and that the hippocampus plays an important role in the pathophysiology of major depressive disorder (Campbell and MacQueen, 2004), the hippocampus has been studied as a major component in the mechanism involved in the treatment of depression. Recently, research has shown that not only the hippocampus but also the prefrontal cortex, thalamus, and hypothalamus are closely related to depression (Pang et al., 2015; Treadway et al., 2015; Kasahara et al., 2016). Stress response is mainly mediated by the hypothalamic-pituitary-adrenal (HPA) axis, and the hypothalamus is a key brain region that has various functions in modulating the endocrine and autonomic nervous systems. The anterior cingulate cortex, amygdala, and hippocampus are anatomically linked to the hypothalamus and midbrain, and interconnected with the prefrontal-limbic network that is strongly related to depressive disorders (Bao et al., 2008; Bennett, 2011).

Previous studies indicated that acupuncture modulates activity within specific brain areas, including the somatosensory-motor cortices, limbic system, basal ganglia and brain stem (Chae et al., 2009; Huang et al., 2011) in healthy subjects. These brain responses reflect acupuncture stimuli-related brain regions. In particular, acupuncture treatment has been reported to cause therapeutic effects by activation of neurons in brain regions such as the somatosensory and motor cortex, prefrontal cortex, cingulate cortex, insular cortex, limbic area, and the hypothalamus (Park et al., 2017a,b). Based on this preliminary report, we found that the therapeutic effect of acupuncture is exerted by a combination of acupuncture stimulated brain regions and disease-related brain regions. Therefore, we broadly selected brain regions that are related to acupuncture stimuli and depression, and then analyzed neural activation in each brain region to identify the acupuncture mechanism by assessing central neural activity. We noted changes in neuronal activity in 15 (2-week CRS model) and 18 (4-week CRS model) of the 30 brain regions. The altered brain area could be divided into the area changed only by AP treatment (at KI10·LR8·LU8·LR4), the area changed only by NP treatment (non-acupoints on the hip), and the area changed by both AP and NP stimulation. The effects of acupuncture therapy included the specific effects of acupuncture as well as nonspecific effects (placebo effect) of acupuncture. Therefore, the brain areas changed by non-acupoint stimulation is thought to be related to the nonspecific effect of acupuncture treatment, and the brain areas changed only by AP treatment (HIP-CA1, HIP-CA3, CC-1, MC-2, and TH-PV in the 2-week CRS model; HIP-CA1, HIP-CA2, CC-1, CC-2, MC-1, MC-2, IC-Gi, IC-Pir, TH-MD, TH-CM, HyTH-DM, HyTH-VM, HyTH-LH, and HyTH-PH in the 4-week CRS model), are considered to be the main brain areas representing the specific effects of acupuncture.

From these results, we could identify that the hippocampus, cingulate cortex, motor cortex, and the thalamus function as important brain regions that exert acupuncture effects. The thalamus acts as a hub that relays information between different subcortical brain areas and the cerebral cortex. It receives sensory signals and sends them to the cortical area (Hwang et al., 2017). The cingulate cortex receives inputs from the thalamus and the neocortex, and projects them to the other brain regions. It integrates the limbic system, which is involved in emotional changes, learning, and memory (Hayden and Platt, 2010). These brain regions are related to both acupuncture signaling transduction and modulating depression. Changes in the motor cortex may be particularly associated with the behavioral improvement of mice.

The neuronal activity in the brain regions associated with the specific effects of acupuncture was prominently increased in the 4-week model as compared to the 2-week model, suggesting that, the longer the treatment period of acupuncture, the more prominent the specific rather than the nonspecific effect was. Moreover, the insular cortex, PAG, and hypothalamic brain areas with increased c-Fos in the 4-week model may be associated with long-term therapeutic effects of acupuncture.

For further mechanistic studies, we observed the expression of serotonin receptors in the core brain regions associated with acupuncture treatment effects. Among the serotonin receptors, 5-HT1A and 5-HT1B receptors have been extensively studied (Savitz et al., 2009). Increased 5-HT1A receptor expression is known to be associated with an improvement in both depressive and anxiety behaviors, while the 5-HT1B receptor plays a role in regulating impulsive behavior, reward, and depression (Heisler et al., 1998; Parks et al., 1998; Knobelman et al., 2001; Parsons et al., 2001). We found that AP treatment increased 5-HT1A receptor expression in the hippocampus, cingulate cortex, motor cortex, thalamus, and the hypothalamus, and 5-HT1B receptor expression in the cingulate cortex, motor cortex, and the thalamus.

**Figure 8:**
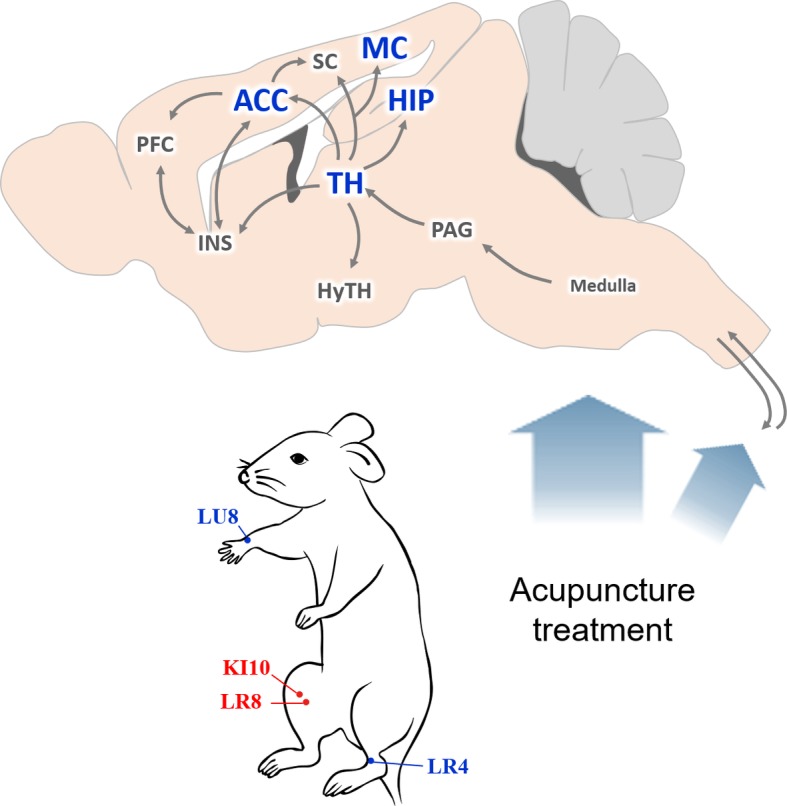
Schematic diagram of the neural mechanism by which acupuncture at AP (KI10·LR8·LU8·LR4) affects depressive-like behavior. A proposed central neural mechanism by which effects of acupuncture needling are exerted. Acupuncture-induced signaling are received by the peripheral nerve endings and are then transmitted to the afferent sensory nervous tract. Brain regions, such as the thalamus, hippocampus, motor cortex, and anterior cingulate cortex are involved in acupuncture-mediated central signals. ACC, anterior cingulate cortex; HIP, hippocampus; HyTH, hypothalamus; INS, insular cortex; MC, motor cortex; PAG, periaqueductal gray; PFC, prefrontal cortex; SC, somatosensory cortex; TH, thalamus.

Based on our results, we hypothesize that a central neural mechanism underlies the effect of KI10·LR8·LU8·LR4 acupuncture treatment (Figure 8). Acupuncture stimulation at KI10·LR8·LU8·LR4 is likely received by peripheral nerve endings, which is then transmitted to the afferent sensory nervous tract. These mechanical signals from the spinal cord might be transmitted to the thalamus, then to the cingulate cortex, somatosensory/motor cortex, insular cortex, and the hippocampus. The thalamus transmits signals to the hypothalamic area, which are then relayed back to the pons and medulla, activating the descending neuronal pathway that provokes the acupuncture effect.

Thus, we found that signal transmission in the brain of a depressive mouse model differed, depending on the acupuncture points used, and the effect of acupuncture correlated with the expression of serotonergic receptors. Although depression studies have mainly focused on the hippocampus, further studies of the prefrontal cortex, thalamus, and hypothalamus are needed. In addition, an analysis of correlations of proteins such as CREB, ERK, and BDNF, in addition to serotonin, is necessary to elucidate the mechanism underlying acupuncture therapeutic effects.

Acupuncture treatment at KI10·LR8·LU8·LR4 was effective in alleviating depressive-like behavior in mice. This therapeutic effect was produced by the modulation of central neural activity and 5-HT1A/B receptor expression in various brain regions of the hippocampus, cortex, thalamus, and the hypothalamus. In future, the effect of the acupoint combination KI10·LR8·LU8·LR4 needs to be clarified in various diseases, and its neurobiological mechanism of acupuncture requires further elucidation to improve our knowledge of the fundamental mechanism.

## Ethics Statement

This study was carried out in accordance with recommendations in the guidelines for the Care and Use of Laboratory Animals of the National Institutes of Health. All experimental protocols used in this study were approved by the Institutional Animal Care and Use Committee (IACUC) at Daejeon University (approval no. DJUARB2016-40).

## Author Contributions

M-JL performed immunostaining, data analysis and drafted the manuscript. J-SR performed animal behavioral tests, immunostaining and data analysis. S-KW performed animal behavioral tests and western blotting. JJ, S-ML, and UN contributed to the conception of the work and assisted with protocol development. J-YP designed the study, coordinated the acquisition of all study data, interpretation of results and writing of the article. All co-authors were involved in critical revision of initial drafts.

### Conflict of Interest Statement

The authors declare that the research was conducted in the absence of any commercial or financial relationships that could be construed as a potential conflict of interest.
