# Supplementary material for: Effects of Acupuncture on Chronic Stress-Induced Depression-Like Behavior and Its Central Neural Mechanism
Source: Front Psychol. 2019 Jul 5;10:1353. doi: 10.3389/fpsyg.2019.01353 (PMC6625224; doi:10.3389/fpsyg.2019.01353)
Supplement: Supplementary file 1 [file Data_Sheet_1.docx]

**Supplementary figures**

**Supplementary figure 1.**

**Changes in body weight during chronic restraint stress and acupuncture treatment.**

**
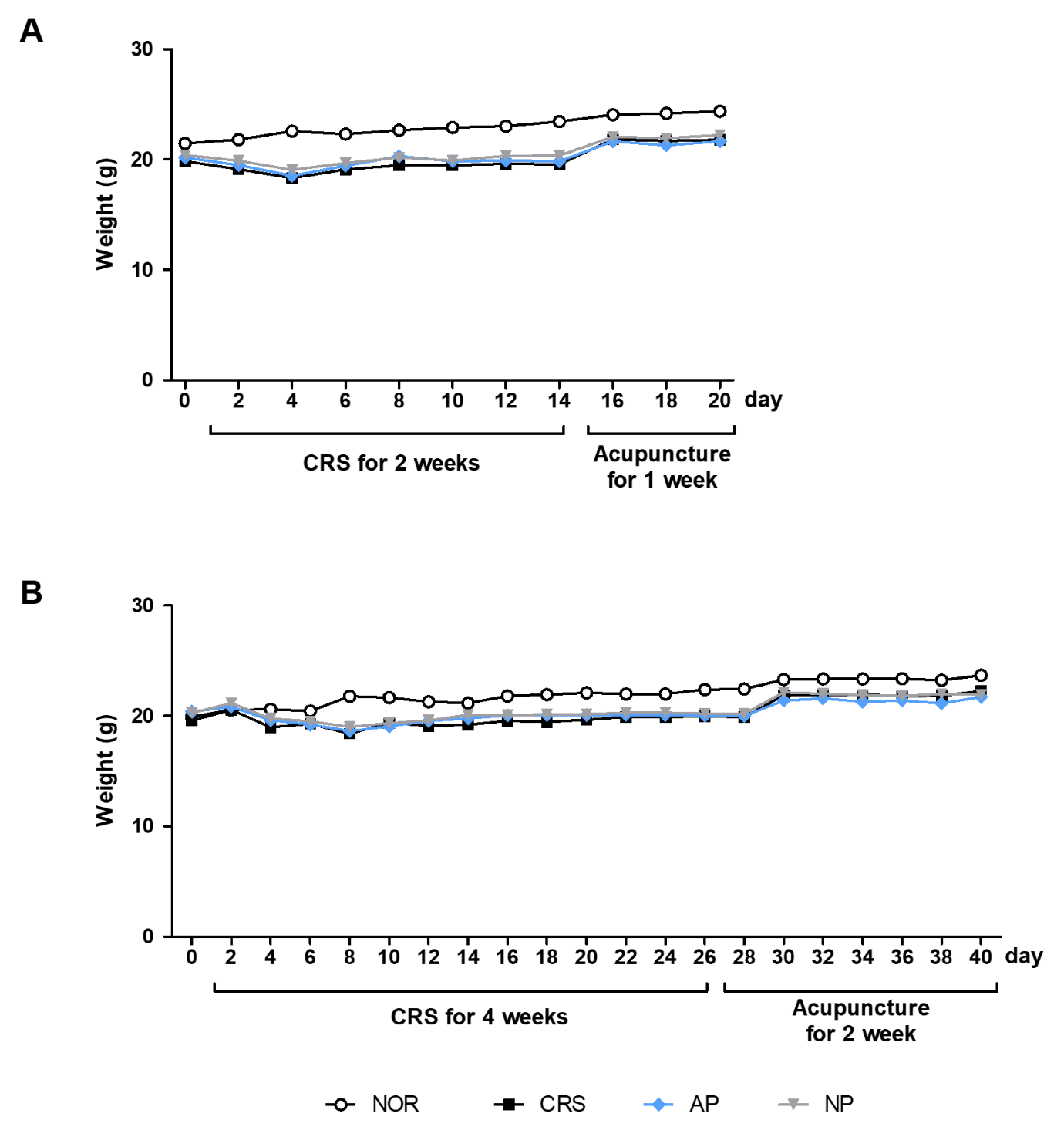
**

(A) Changes in body weight in 2-weeks chronic restraint stress model. (B) Changes in body weight in 4-weeks chronic restraint stress model. NOR: normal, AP: CRS and acupuncture treatment at KI10·LR8·LU8·LR4; NP: CRS and acupuncture treatment at non-acupoints on the hips.

**Supplementary figure 2.**

**Changes in c-Fos activation after acupuncture treatment at AP (KI10·LR8·LU8·LR4) in the hippocampus, thalamus, hypothalamus, and cingulate cortex.**

**
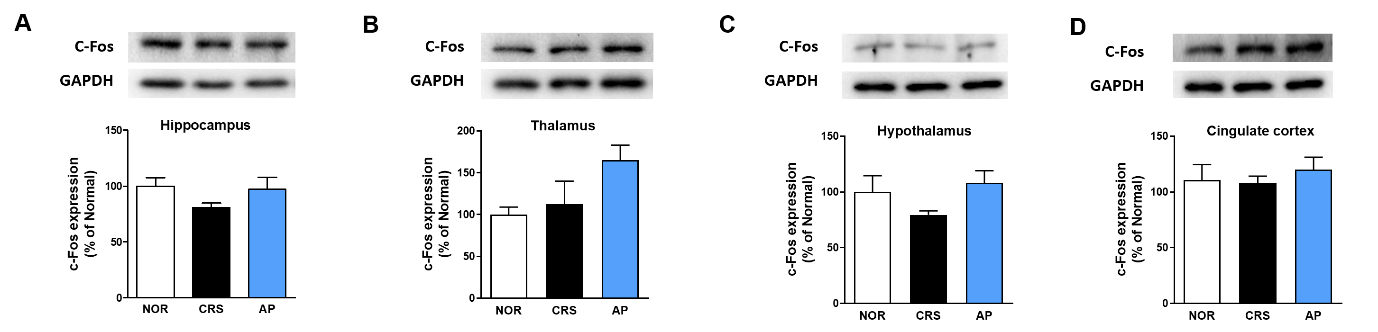
**

Acupuncture treatment at AP (KI10·LR8·LU8·LR4) slightly increased the c-Fos activation in hippocampus, thalamus, hypothalamus, and cingulate cortex, but this increase was not statistically significant. NOR: normal, AP: CRS and acupuncture treatment at KI10·LR8·LU8·LR4. One-way ANOVA followed by the Newman-keuls test. Error bars indicate SEM.
